# Transnasal Videoendoscopy for Preoperative Airway Risk Stratification: Development and Validation of a Multivariable Risk Prediction Model

**DOI:** 10.1213/ANE.0000000000006418

**Published:** 2023-04-19

**Authors:** Alexander Barclay-Steuart, Hanna L. Großhennig, Phillip Sasu, Viktor A. Wünsch, Rupert Stadlhofer, Joanna Berger, Maria Stark, Susanne Sehner, Christian Zöllner, Martin Petzoldt

**Affiliations:** From the * Department of Anesthesiology, Center of Anesthesiology and Intensive Care Medicine, University Medical Center Hamburg-Eppendorf, Hamburg, Germany; † Department of Otorhinolaryngology; ‡ Institute of Medical Biometry and Epidemiology, University Medical Center Hamburg-Eppendorf, Hamburg, Germany.

## Abstract

Transnasal flexible videoendoscopy (TVE) of the larynx is a standard of care for the detection and staging of pharyngolaryngeal lesions in otorhinolaryngology. Patients frequently present with existing TVE examinations before anesthesia. Although these patients are considered high risk, the diagnostic value of TVE for airway risk stratification is currently unknown. How can captured images or videos be used for anesthesia planning, and which lesions are most concerning? This study aimed to develop and validate a multivariable risk prediction model for difficult airway management based on TVE findings and to determine whether the discrimination of the Mallampati score can be improved by adding this new TVE model.

**METHODS:**

This retrospective single-center development and validation study assessed 4021 patients who underwent 4524 otorhinolaryngologic surgeries at the University Medical Centre Hamburg-Eppendorf between January 1, 2011, and April 30, 2018, with electronically stored TVE videos and included 1099 patients who underwent 1231 surgeries. TVE videos and anesthesia charts were systematically reviewed in a blinded fashion. The Least Absolute Shrinkage and Selection Operator (LASSO) regression analysis was used for variable selection, model development, and cross validation.

**RESULTS:**

The prevalence of difficult airway management was 24.7% (304/1231). Lesions at the vocal cords, epiglottis, or hypopharynx were not selected by the LASSO regression, while lesions at the vestibular folds (ß-coefficient 0.123), supraglottic region (ß-coefficient 0.161), arytenoids (ß-coefficient 0.063), and viewing restrictions on the rima glottidis that cover ≥50% of the glottis area (ß-coefficient 0.485) and pharyngeal secretion retention (ß-coefficient 0.372) were relevant risk factors for difficult airway management. The model was adjusted for sex, age, and body mass index. The area under the receiver operating characteristic curve (95% confidence interval) of the Mallampati score was 0.61 (0.57–0.65) and 0.74 (0.71–0.78) of the TVE model combined with Mallampati (*P* < .001).

**CONCLUSIONS:**

Stored images and videos from TVE examinations can be reused for the purpose of predicting risk associated with airway management. Vestibular fold, supraglottic, and arytenoid lesions are most concerning, especially if they are accompanied by secretion retention or restrict the glottic view. Our data indicate that the TVE model improves discrimination of the Mallampati score and might, therefore, be a useful addition to traditional bedside airway risk examinations.

**KEY POINTS**

- **Question:** Can stored images and videos from preoperative transnasal flexible videoendoscopy (TVE) examinations be reused for the purpose of predicting risk associated with airway management in patients undergoing otorhinolaryngologic surgery?
- **Findings:** We developed a multivariable Least Absolute Shrinkage and Selection Operator (LASSO) regression model (TVE model) that identified the most concerning lesions in existing TVE examinations and substantially improved discrimination when added to the Mallampati score.
- **Meaning:** Our data indicate that the TVE model might be a useful addition to traditional bedside airway risk examinations and identified vestibular fold, supraglottic, and arytenoid lesions as the most concerning findings, especially if they are accompanied by retention of secretions or restrict the glottic view.

Airway management problems are one of the main reasons for anesthesia-related adverse events.^1–3^ Transnasal flexible videoendoscopy (TVE) of the upper aerodigestive tract (also known as “nasendocopy”) is a standard of care for the detection, classification, and staging of pharyngolaryngeal lesions in otorhinolaryngology.^4^ Patients with suspected or known pharyngolaryngeal lesions, such as tumors, hyperplasia, edema, abscesses, or other space-consuming lesions, often require surgery or diagnostic procedures. Hence, patients frequently present with existing TVE examinations before general anesthesia.

Current guidelines recommend that airway evaluation may include preoperative bedside endoscopy.^5^ However, although patients with pharyngolaryngeal lesions are at high risk for difficult airway management,^6–10^ the diagnostic value of these pathological TVE findings for predicting risk associated with airway management is still unknown. An evidence-based score, assessment tool, or structured workflow is lacking. At present, it remains unclear how existing TVE examinations could be used for anesthesia planning and integrated into existing concepts for risk stratification, preinduction strategies, and decision-making, particularly for or against awake tracheal intubation.^8,10,11^ It remains unclear which findings, tumor locations, spread, and size are most concerning and whether pathological TVE findings can improve discrimination if added to traditional bedside risk assessment tests. Captured images and videos from routine otorhinolaryngological TVE examinations could be shared with and reused by anesthesiologists for the purpose of predicting risk associated with airway management; consequently, quality and patients’ safety could be improved without additional measures or expense. At present, neither the architecture of pharyngolaryngeal lesions nor TVE findings are represented in traditional bedside airway risk prediction scores.^12–16^ It is not known whether systematic preoperative mapping of pharyngolaryngeal lesions and structured scoring of TVE findings improve prediction of risk associated with airway management.

This retrospective cohort study aimed to develop and validate a multivariable least absolute shrinkage and selection operator (LASSO) regression model for the prediction of difficult airway management based on TVE findings. A secondary aim was to determine whether discrimination of the Mallampati score^17^ can be improved by adding this new TVE model.

## METHODS

This multivariable prediction model development and validation study was conducted in accordance with the Declaration of Helsinki. The design and reporting are adapted from the Transparent Reporting of a Multivariable Prediction Model for Individual Prognosis or Diagnosis (TRIPOD) statement.^18,19^ The statistical analysis and reporting refer to the Statistical Analysis and Reporting Guidelines for CHEST.^20^ The Ethics Committee of the Medical Association of Hamburg approved this retrospective study and waived the need to obtain written informed consent for collection, analysis, and publication of data (WF-022/18, April 16, 2018, Chairman/Professor Dr Stahl).

### Patient Selection and Data Collection

We used an anonymized data set for our statistical analysis, which was collected for internal quality assessment. This anonymized data sample was originated from adult patients who underwent otorhinolaryngologic surgery at the University Medical Centre Hamburg-Eppendorf between January 1, 2011, and April 30, 2018. Only cases with preoperative TVE examinations not older than 30 days before surgery were selected. Recordings from repeated anesthetics and the corresponding TVE data and videos were only included in the analysis if patients had a new TVE examination not older than 30 days and still met all eligibility criteria. Anesthesia charts were systematically reviewed for the primary and secondary outcomes by 1 assessor (H.L.G.) and supervised by 2 anesthetists (A.B.S. and M.P.). Assessors were blinded to the TVE findings. Borderline findings were reviewed by all 3 assessors (H.L.G., A.B.S., and M.P.), discrepancies were discussed, and a consensus agreement regarding the question of whether the patient met the outcome definition was reached in each case. TVE videos were reviewed using a predefined registration sheet including a systematic mapping of lesions by 1 assessor (H.L.G.) supervised by 2 otorhinolaryngologists (R.S. and J.B.). Assessors were blinded to the results of the primary and secondary outcome assessments from the anesthesia charts (anonymized video analysis).

Unclear or borderline findings in the TVE videos were reviewed by all 3 assessors (H.L.G., R.S., and J.B.), discrepancies were discussed, and a consensus agreement was reached in each case. To determine interobserver reliability, 92 consecutive videos were independently analyzed by 3 assessors (H.L.G., R.S., and J.B.) within a structured protocol, blinded to each other’s ratings.

### Eligibility Criteria

**Figure 1.**
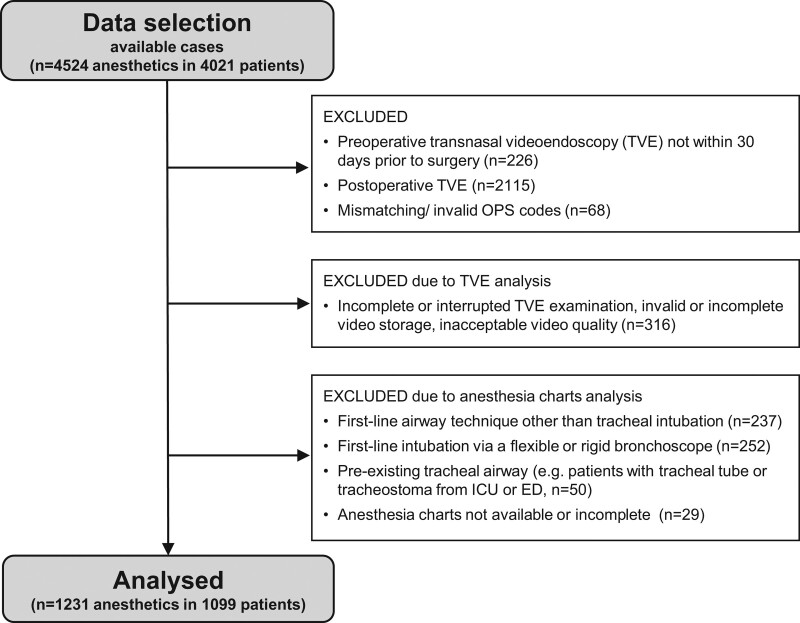
Study flow.

The study flow and data selection process are given in Figure 1. Inclusion criteria were: age 18 years or older, otorhinolaryngologic surgery with general anesthesia, tracheal intubation facilitated either by direct laryngoscopy or videolaryngoscopy as first-line technique, and a TVE recording within 30 days before surgery.

**Table 1.** Outcome Definition

Primary outcome measure	Definition
Difficult airway management	Composite of difficult tracheal intubation or difficult facemask ventilation
**Secondary outcome measures**	**Definition**
Difficult tracheal intubation	Clearly documented difficult tracheal intubation, failed first-line intubation method with transition to a rescue airway technique, documented multiple tracheal intubation attempts, or clearly noted impaired laryngeal view (eg, Cormack-Lehane grade III or IV)
Multiple intubation attempts	Clearly documented ≥2 intubation attempts by the same or a different operator
Transition to a rescue technique	Clearly documented conversion to a different tracheal intubation technique (eg videolaryngoscopy after failed direct laryngoscopy or bronchoscopy after failed videolaryngoscopy or direct laryngoscopy
Cormack-Lehane class (I–IV)	Documented best laryngeal view on the larynx as graded by the Cormack-Lehane classification
Optimization maneuvers	For example BURP, OELM, bougie application, and direct epiglottis lifting
Difficult facemask ventilation	Clearly documented
Impossible facemask ventilation	Clearly documented
Airway-related adverse events	Bronchospasm, laryngospasm, hypoxia with SpO_2_ <90%, soft tissue injuries, postintubation laryngeal edema requiring corticosteroids or esophageal intubation

Definition of primary and secondary outcome measures derived from systematic chart review.

Abbreviations: BURP, backward upward rightward pressure; OELM, optimum external laryngeal manipulation; SpO_2_, peripheral oxygen saturation.

Exclusion criteria were: mismatching, invalid or inconclusive operation and procedure classification system codes (OPS codes), incomplete or interrupted TVE examinations, invalid or incomplete video storage, inacceptable video quality, preexisting tracheostomy, a preexisting tracheal tube (eg, patients from an intensive care unit), as well as unavailable or incomplete anesthesia charts.

### Outcome Measures

The primary and secondary outcome measures derived from the systematic chart review are given in Table 1.

### Predictor Variables

Potentially eligible covariables (candidate predictors) for the primary outcome measure were identified by literature research, previous studies, and clinical considerations:

1. Pharyngolaryngeal lesions identified in the TVE videos (multiple choices possible):
  a) Hypopharynx lesions that consume ≥25% of the transverse diameter (yes/no)
  b) Supraglottic region lesions (yes/no)
  c) Arytenoid lesions (yes/no)
  d) Vocal cord lesions (yes/no)
  e) Vestibular fold lesions (yes/no)
  f) Epiglottis lesions (yes/no)
2. Viewing restrictions on the rima glottidis due to lesions (none/relevant viewing restriction that cover less than half of the glottis cross-sectional area/more than half of the glottis area)
3. Secretion retention (yes/no)
4. Sex (male/female)
5. Age (years)
6. Body mass index (BMI) (kg·m^−2^)

The Mallampati score (modification of Samsoon and Young, I–IV^17^) was used as a comparator.

### Sample Size

We used the approach proposed by Riley et al^21^ to verify the required sample size for model development after completion of data sampling using 3 criteria. First, the sample size should ensure an accurate estimate of the overall outcome risk. Based on literature review and our own in-house data,^6,7,9,22^ we assumed a primary end point prevalence of 0.15 in patients undergoing otorhinolaryngologic surgery and a margin of error ≤0.05. Second, the sample size should lead to a shrinkage of predictor effects of 10%. Third, the sample size should lead to a small optimism in apparent model fit. To calculate the sample size, we assumed a C-statistic of 0.7 and 12 candidate predictors to be appropriate. The sample size for each criterion was calculated, and the maximum sample size was chosen. A required sample size of 981 patients was approximated.

### Descriptive Statistics

Sample characteristics are given as absolute and relative frequencies or mean (standard deviation), whichever was appropriate. We used Fleiss’ kappa to calculate the agreement between the 3 independent observers regarding the ratings of the TVE videos.

Statistical analysis was performed using SPSS statistics version 25 (IBM Inc) and R version 4.0.2 (R Foundation for Statistical Computing).

### Multivariable Model Development, Validation, and Performance

For model development and internal validation, we performed LASSO regression using all 12 identified candidate predictors. For model development, zero points of metric valuables were shifted based on a data-driven approach (age) and on the World Health Organization classification for BMI.^23^ In the first step, a 10-fold cross-validation was used to determine the shrinkage parameter λ. In the second step, based on the resulting largest λ with a cross-validated error within 1 standard error of the cross-validated error for the minimal lambda (λ_1se_), a LASSO regression—again with a 10-fold cross-validation—was calculated to estimate the shrunken β-coefficients. The second step was repeated 20 times. For each of these 200 validation cohorts, the area under the receiver operating characteristic curve (AUC) was calculated. We report the cross-validated mean AUC (cvAUC) with resulting standard deviation. The final coefficients result from the best-fitting model (highest AUC). Coefficients from the best-fitting LASSO regression model, which were not shrunk to zero, were considered predictors in the final multivariable LASSO regression model (TVE model).

### Model Comparison

To determine whether the new TVE model improves discrimination when added to a traditional bedside difficult airway risk assessment test, we calculated the AUC with 95% confidence interval (95% CI) of the Mallampati score (modification of Samsoon and Young, I–IV^17^) and of the Mallampati score combined with the TVE model. We used the nonparametric approach by DeLong et al^24^ to compare the AUCs between models. For this analysis, we present *P* values without correction for multiplicity.

### Score Development and Specification

For the development of a clinically applicable simplified TVE score, we weighted each predictor variable that was not shrunk to zero in the LASSO regression; for this purpose, β-coefficients were multiplied by 10. Metric variables were again multiplied by 10 to obtain score points per 10 years and per 10 kg m^−2^ and categorized. Values were rounded to provide integer score points. Based on our findings, age was categorized in risk groups (50–59, 60–69, and ≥70 years), and BMI was dichotomized (at least moderate obesity with BMI ≥35 kg m^−2^ [y/n]^23^) after the development of the TVE model.

To assess the discrimination of the simplified TVE score, we calculated the AUC (95% CI). To further assess the prediction accuracy of the simplified TVE score, we calculated the index of prediction accuracy (IPA).^25^

We calculated the sensitivity, specificity, positive predictive value (PPV), and negative predictive value (NPV) for the final simplified TVE score. We used sensitivity and specificity from a utility-based perspective to determine the optimal decision thresholds. We claimed that a “screening cut-off value” should ensure a sensitivity above 60% and that an additional “diagnostic cut-off value” should ensure a specificity above 80%.

## RESULTS

We identified 4021 patients who had undergone 4524 otorhinolaryngologic surgeries in general anesthesia at the University Medical Centre Hamburg-Eppendorf within the study period whose electronic patient records included a TVE video (Figure 1). We identified 1231 anesthetic cases in 1099 patients, who fulfilled all eligibility criteria. Baseline characteristics are given in Table 2.

**Table 2.** Study Cohort Baseline Characteristics

Variables	Study cohort (N = 1231)
Age, y	56.9 ± 16.3
BMI, kg m^−2^	25.6 ± 4.8
Sex	
Male	829 (67.3)
Female	402 (32.7)
ASA physical status classification	
I	175 (14.2)
II	582 (47.3)
III	450 (36.6)
IV	24 (1.9)
Mallampati score	
I	479 (40.6)
II	536 (45.4)
III	150 (12.7)
IV	16 (1.4)
Surgical procedures (various combinations possible)^a^	
Microlaryngoscopy	662 (53.8)
Laryngopharyngeal, tracheal	489 (39.7)
Endocrine glands	29 (2.4)
Ear	103 (8.4)
Nose and sinuses	264 (21.4)
Face and oral cavity	285 (23.2)

Values are mean ± SD or number (proportion), whichever is appropriate; the dataset of this analysis is complete (n = 1231) with the exception of the Mallampati score (50 missing values) and BMI (1 missing value).

Abbreviations: ASA, American Society of Anesthesiologists; BMI, body mass index; SD, standard deviation.

a Type of surgery was categorized: “endocrine glands” subsumes thyroid and parathyroid surgery, “ear” subsumes operations on the inner and middle ear, acoustic meatus and conch; “face and oral cavity” subsumes operations of the tongue, salivary glands or their excretory ducts, face, and oral cavity.

**Table 3.** Outcome Measures

Variables	Overall (N = 1231)	With difficult airway management (n = 304)	Without difficult airway management (n = 927)
First-line intubation technique			
Direct	1121 (91.1)	238 (78.3)	883 (95.3)
Videolaryngoscopy	110 (8.9)	66 (21.7)	44 (4.7)
Primary outcome measure			
Difficult airway management	304 (24.7)	304 (100)	–
Secondary outcome measures			
Difficult tracheal intubation	261 (21.2)	261 (85.9)	–
Multiple intubation attempts	81 (6.6)	81 (26.6)	–
Transition to a rescue technique	61 (5.0)	61 (20.1)	–
Videolaryngoscopy	58 (4.7)	58 (19.1)	–
Bronchoscopic intubation	3 (0.2)	3 (1.0)	–
Cormack-Lehane class			
I	714 (64.2)	45 (16.7)	669 (79.5)
II	286 (25.7)	113 (41.9)	173 (20.5)
III	96 (8.6)	96 (35.6)	–
IV	16 (1.4)	16 (5.9)	–
Optimization maneuvers			
Bougie application	30 (2.4)	30 (9.9)	–
BURP/OELM	104 (8.4)	82 (27.0)	22 (2.4)
Direct epiglottis lifting	8 (0.6)	7 (2.3)	1 (0.1)
Facemask ventilation			
Difficult	69 (5.6)	69 (22.7)	–
Impossible	3 (0.2)	3 (1.0)	–
Airway-related adverse events	19 (1.5)	17 (5.6)	2 (0.2)
Larynx edema^a^	5 (0.4)	5 (1.6)	–
Bronchospasm	1 (0.1)	0	1 (0.1)
Hypoxia (SpO_2_ < 90%)	3 (0.2)	3 (1.0)	–
Soft tissue injury	10 (0.8)	9 (3.0)	1 (0.1)

Values are number (proportion), and the dataset of this analysis is complete (N = 1231) except for the Cormack-Lehane classification (119 missing values, n = 1112, n = 270 with and n = 842 without the primary outcome); the primary outcome measure "difficult airway management" is a composite of difficult tracheal intubation and difficult facemask ventilation.

Abbreviations: BURP, backward upward rightward pressure; OELM, optimum external laryngeal manipulation.

a With consecutive systemic application of corticosteroids.

**Table 4.** Multivariable LASSO Regression Model (TVE Model) and Development of the TVE Score

	TVE model	TVE score	
Predictor variables TVE model	β-coefficient (with λ_1se_ = 0.0326)	ß-coefficients (10-fold)	Final TVE score
Intercept	−2.577	−25.77	
Laryngopharyngeal lesions			
Hypopharynx^a^	Shrunken to zero		
Supraglottic region	0.161	1.61	2 pts
Arytenoids	0.063	0.63	1 pt
Vocal cords	Shrunken to zero		
Vestibular folds	0.123	1.23	1 pt
Epiglottis	Shrunken to zero		
Viewing restriction on rima glottidis			
Relevant, covers <50% of the glottis area	Shrunken to zero		
Covers ≥50% of the glottis area	0.485	4.85	5 pts
Pharyngeal secretion retention	0.372	3.72	4 pts
Sex, male	0.082	0.82	1 pt
Age, y (zero point shifted to 40 y)^b^	0.022	0.22 (2 per 10 y increase)	
		50–59 y	2 pts
		60–69 y	4 pts
		≥70 y	6 pts
BMI, kg m^−2^ (zero point shifted to 18.5 kg m^−2^)^b^	0.004	0.04 (0.4 per 10 kg m^−2^ increase)	
		At least moderate obesity (≥35 kg m^−2^)	1 pt
Total score range			0–21 pts
cvAUC (SD) of the TVE model	0.70 (0.05)		
AUC (95% CI) of the TVE score		0.71 (0.67–0.74)	

Reported are: λ_1se_-shrinkage parameter determined by a 10-fold cross-validation, β-coefficients derived from the LASSO regression model with the highest AUC, the mean of 20 times repeated 10-fold cvAUC with SD; β-coefficients that were not shrunk to zero were considered relevant predictors.

Abbreviations: BMI, body mass index; cvAUC, cross-validated area under the curves; LASSO, least absolute shrinkage and selection operator; SD, standard deviation; TVE, transnasal videoendoscopy of the larynx.

a Hypopharynx lesions were accounted if they consumed more than a quarter of the transverse diameter.

b For model development, zero points of metric valuables were shifted based on a data-driven approach for age and based on the World Health Organization classification for BMI (underweight BMI <18.5 kg m^−2^, overweight BMI 25–29.9 kg m^−2^, obesity class I BMI 30–34.9 kg m^−2^, class II BMI 35–39.9, class III BMI ≥40 kg m^−2^); for the TVE score, β-coefficients were multiplied by 10 and rounded to integer numbers; metric variables were categorized after model development.

The primary outcome measure difficult airway management was found in 24.7% (304/1231), difficult tracheal intubation in 21.2% (261/1231), and a difficult facemask ventilation in 5.6% (69/1231) of the cases (Table 3). In 91.1% (1121/1231) of the cases, direct laryngoscopy was used as the first-line intubation technique. The primary tracheal intubation technique failed in 5.0% (61/1231) and either videolaryngoscopy (4.7% [58/1231]) or intubation with a bronchoscope (0.2% [3/1231]) was used as rescue techniques.

### Multivariable Model Development, Validation, and Performance

For the development of the multivariable LASSO regression model (TVE model), 12 candidate predictors were selected by clinical considerations, literature search, and previous studies. Lesions at the vocal cords, epiglottis, or hypopharynx were not selected by the LASSO regression (shrunk to zero), while lesions at the vestibular folds (ß-coefficient 0.123), supraglottic region (ß-coefficient 0.161), and arytenoids (ß-coefficient 0.063) were found to be relevant risk factors for difficult airway management in the LASSO regression (Table 4). Furthermore, pharyngeal secretion retention (ß-coefficient 0.372), restricted view on the rima glottidis due to lesions that cover more than half of the glottis cross-sectional area (ß-coefficient 0.485), age, male sex, and BMI were found to be relevant risk factors that were not shrunk to zero in the LASSO regression. The final TVE model encompasses 8 important covariables, while 4 predictors were shrunk to zero (Table 4). The mean cvAUC of all 200 validation cohorts was 0.70 (standard deviation 0.05).

### Model Comparison

The AUC (95% CI) of the Mallampati score was 0.61 (0.57–0.65) and 0.74 (0.71–0.78) for the TVE model, combined with the Mallampati score with *P* < .001.

### Simplified TVE Score

**Figure 2.**
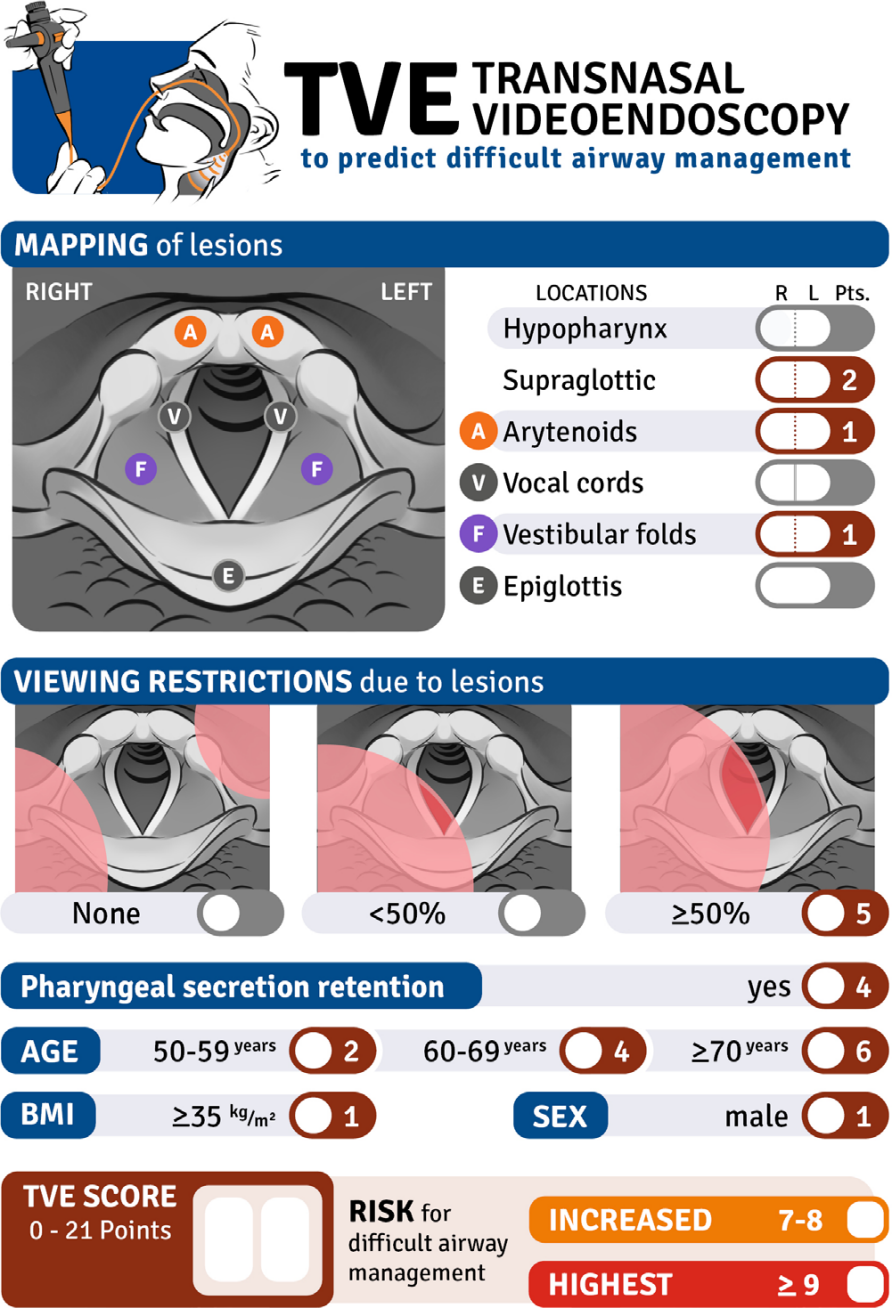
Structured assessment, recording, and scoring of TVE findings. Illustration by Rasmus Borkamp, Hamburg, Germany.

The final simplified TVE score has a range between 0 and 21 points (Figure 2). The AUC of the TVE score based on the complete training set is 0.71 (0.67–0.74). We calculated the IPA of the TVE score based on the complete training set that is 0.01%.^25^

The optimal decision thresholds were determined from a utility-based perspective using a “screening cut-off value” (≥7 points) with sensitivity above 60% and a “diagnostic cut-off value” (≥9 points) with a specificity above 80%, resulting in a risk ranking: 7–8 points “increased risk” and ≥9 points “highest risk” (Supplemental Digital Content, Table S1, http://links.lww.com/AA/E231). Based on these thresholds, 19.2% of the cases in our cohort were classified as “increased risk” (difficult airway management prevalence 30.6%) and 20.9% as “highest risk” (prevalence 45.4%) for difficult airway management.

Fleiss’ kappa analysis revealed moderate agreement between observers for the classification of “increased risk” (0.532) and substantial agreement for the classification of the “highest risk” (0.621) with the simplified TVE score.^26^

## DISCUSSION

Transnasal flexible endoscopy is a standard-of-care diagnostic measure for patients with hoarseness or with suspected laryngeal cancer.^4,27^ Many of these patients require general anesthesia for subsequent surgery or diagnostics such as microsurgery thereafter. When we conducted the TVE study, we discussed how to use stored images or videos from TVE examinations for prediction of risk associated with airway management and anesthesia planning. From the patients’ viewpoint, the benefit is obvious; physicians simply have to reuse existing data from previous examinations without exposing patients to any additional risk, inconvenience, or time-consuming additional diagnostic measures. Quality and patients’ safety can be improved without causing additional expense, while health care resources can be preserved.

Patients with pharyngolaryngeal lesions are at high risk for difficult tracheal intubation^6–10^ with an expected incidence up to 28%,^7^ which is attributed to the size, location, bleeding tendency, risk of swelling, and impaired view during airway management.^6,7,28^ Predictive tests, developed in cross-sectional surgical populations with a low average risk, are suspected to have poor discrimination in patients with laryngopharyngeal disease, as they are not tailored to these patients.^7,29^ A widely accepted tool for classification of these findings (eg, in the electronic health record) has not yet been established in otolaryngology.^30–32^

We developed and validated a multivariable LASSO regression model for the prediction of difficult airway management—the TVE model—which can be used by anesthesiologists or otorhinolaryngologists as a tool for preoperative risk stratification.

Male sex, moderate to severe obesity (BMI ≥35 kg m^−2^), and increased age were important risk factors for difficult airway management and were used for adjustment of the multivariable TVE model. Supraglottic, arytenoid, and vestibular fold lesions were the most concerning locations of pharyngolaryngeal lesions, while lesions of the vocal cords, epiglottis, and hypopharynx were not concerning. Further findings of concern were viewing restrictions on the glottis that cover more than the half of the glottis cross-sectional area (typically caused by space-consuming lesions) and pharyngeal secretion retention (eg, pooled secretion in the pyriform sinus) due to dysphagia.

The most recommended bedside difficult airway risk assessment tests are the upper lip bite test (ULBT) and Wilson score.^12,15^ However, both scores rely solely on functional and anatomic assessments of the head and neck regions, such as mouth opening, mandibular jaw, and cervical spine mobility, while laryngopharyngeal lesions are ignored and thus present a fateful “blind spot” in current bedside risk assessment tests. Our findings suggest that TVE has a great potential to close this gap.

We found that the TVE model substantially improved discrimination when added to the Mallampati score. The simplified TVE score is intended to be embedded in existing risk scores and might be a useful addition to traditional bedside difficult airway risk assessment tests.^12,13,15^

The notion of a preoperative risk prediction tool based on existing TVE examinations, stored images, or videos is novel. However, some previous studies have investigated the value of indirect transoral laryngoscopy^33^ or TVE examinations,^11,34,35^ ordered solely for the purpose of airway risk assessment. The risks, efforts, and expenses of this additional minimal invasive diagnostic measure must be weighed against its possible benefit. A previous study found that a visual inspection of airway pathology by TVE relevantly triggered the decision for or against awake tracheal intubation^11^; however, it remains unclear if this changed the outcome. Which concrete objective findings should individual decision-making rely on? Guo et al^35^ found that preoperative TVE, that orientated on the number of visible subglottic tracheal rings, had higher discrimination than the Mallampati or Wilson scores to predict difficult intubation. However, individuals with laryngeal lesions were excluded.

Gemma et al^34^ used TVE for an anatomic–functional assessment of the laryngopharyngeal region, similar to the modified Cormack–Lehane classification, in 169 patients undergoing ear, nose, or throat (ENT) surgery. Individuals with pharyngolaryngeal neoplasm or previous radiation therapy were excluded. They found an improved prediction of their “endoscore” in addition to a routine bedside evaluation to predict difficult intubation.

Although data regarding preoperative TVE are limited, the current 2022 American Society of Anesthesiologists (ASA) guidelines recommended that airway evaluation may include preoperative bedside endoscopy.^5^ The 2021 Canadian Airway Focus Group recommended that nasal endoscopy immediately before airway management in patients with known or suspected obstructing glottic or supraglottic airway pathology may be helpful.^36^ Our findings clearly underline these recommendations and provide additional insight into how to interpret findings and to implement a structured assessment and diagnostic workflow.

Although videolaryngoscopy has revolutionized airway management,^37^ TVE has been studied almost exclusively in conjunction with conventional, direct laryngoscopy, while data in patients undergoing videolaryngoscopy are very limited.^38^ As improvement of the laryngeal view during intubation is the most obvious domain of videolaryngoscopy, the question arises as to which role is played by TVE if videolaryngoscopy is globally available at the bedside. How informative and predictive is preoperative TVE in conjunction with subsequent videolaryngoscopy? In our study, videolaryngoscopy was used as a first-line technique in 110 patients and further as a rescue technique in 58 cases; however, further studies are needed to determine the diagnostic value of TVE in the era of videolaryngoscopy.

Our data only reflect single-center experiences, and caution should be exercised should our results be generalized. In particular, tracheal intubation and TVE techniques may differ between institutions. Further external validation of the TVE model could reinforce our findings. As it is well-known that converting a regression model to an integer score to improve clinical applicability discards useful information and alters calibration, the unsimplified coefficient model should be used for risk prediction whenever possible.^39^ It should be considered that our study cohort comprises patients in whom TVE was indicated for otolaryngological reasons; hence, the prevalence of difficult airway management in our cohort was high, and even individuals with a low TVE score were at higher risk than an average person. Although TVE images and videos were not routinely available for the anesthetists in the study period, the retrospective nature of this study is a well-recognized limitation and a potential source for selection and diagnostic review bias.^40^

## CONCLUSIONS

Pharyngolaryngeal lesions still represent a faithful “blind spot” in traditional bedside airway risk assessment tests. Captured images and videos from existing TVE examinations can be reused by anesthesiologists or otorhinolaryngologists to stratify the risks associated with airway management. While supraglottic, arytenoid, and vestibular fold lesions were concerning, vocal cord, epiglottis, and hypopharynx lesions were not. Relevant viewing restrictions on the glottis and pharyngeal secretion retention were associated with difficult airway management. We developed and validated a novel TVE model that substantially improved discrimination when added to the Mallampati score, and is a useful addition to traditional bedside airway risk examinations.

## DISCLOSURES

**Name**: Alexander Barclay-Steuart, MD, MBA.

**Contribution**: This author helped design the study, conduct the study, analyze and interpret the data, write the manuscript, and read and approve the final manuscript.

**Conflicts of Interest**: None.

**Name**: Hanna L. Großhennig.

**Contribution**: This author helped design the study, conduct the study, analyze and interpret the data, perform the literature review and draft the initial manuscript, and read and approve the final manuscript.

**Conflicts of Interest**: None.

**Name**: Phillip Sasu, MD.

**Contribution**: This author helped interpret the data, review the manuscript, edit and modify the drafts, and read and approve the final manuscript.

**Conflicts of Interest**: None.

**Name**: Viktor A. Wünsch, MD.

**Contribution**: This author helped interpret the data, review the manuscript, edit and modify the drafts, and read and approve the final manuscript.

**Conflicts of Interest**: None.

**Name**: Rupert Stadlhofer, MD.

**Contribution**: This author helped conduct the study, interpret the data, review the manuscript, and read and approve the final manuscript.

**Conflicts of Interest**: None.

**Name**: Joanna Berger, MD.

**Contribution**: This author helped conduct the study, interpret the data, review the manuscript, and read and approve the final manuscript.

**Conflicts of Interest**: None.

**Name**: Maria Stark, MSc.

**Contribution**: This author helped design the study, analyze and interpret the data, perform all statistical analyses including sample size calculation, create the figures, review the manuscript, and read and approve the final manuscript.

**Conflicts of Interest**: None.

**Name**: Susanne Sehner, MSc.

**Contribution**: This author helped design the study, analyze and interpret the data, perform all statistical analyses including sample size calculation, create the figures, review the manuscript, and read and approve the final manuscript.

**Conflicts of Interest**: None.

**Name**: Christian Zöllner, MD.

**Contribution**: This author helped review the manuscript, edit and modify the drafts, and read and approve the final manuscript.

**Conflicts of Interest**: None.

**Name**: Martin Petzoldt, MD.

**Contribution**: This author helped design the study, conduct the study, analyze and interpret the data, perform the literature review, and write and revise the final manuscript.

**Conflicts of Interest**: M. Petzoldt received a research grant awarded by Verathon Inc, Bothell, WA.

**This manuscript was handled by:** Narasimhan Jagannathan, MD, MBA.

## Supplementary Material



## References

[1] Cook TM Woodall N Frerk C; Fourth National Audit Project. Major complications of airway management in the UK: results of the Fourth National Audit Project of the Royal College of Anaesthetists and the Difficult Airway Society. Part 1: anaesthesia. Br J Anaesth. 2011;106:617–631. 21447488 10.1093/bja/aer058

[2] Huitink JM Lie PP Heideman I. A prospective, cohort evaluation of major and minor airway management complications during routine anaesthetic care at an academic medical centre. Anaesthesia. 2017;72:42–48. 27665740 10.1111/anae.13640

[3] Joffe AM Aziz MF Posner KL. Management of difficult tracheal intubation: a closed claims analysis. Anesthesiology. 2019;131:818–829. 31584884 10.1097/ALN.0000000000002815 PMC6779339

[4] Jones TM De M Foran B Harrington K Mortimore S. Laryngeal cancer: United Kingdom national multidisciplinary guidelines. J Laryngol Otol. 2016;130:S75–S82. 27841116 10.1017/S0022215116000487 PMC4873912

[5] Apfelbaum JL Hagberg CA Connis RT. American Society of Anesthesiologists practice guidelines for management of the difficult airway. Anesthesiology. 2022;136:31–81. 34762729 10.1097/ALN.0000000000004002

[6] Arne J Descoins P Fusciardi J. Preoperative assessment for difficult intubation in general and ENT surgery: predictive value of a clinical multivariate risk index. Br J Anaesth. 1998;80:140–146. 9602574 10.1093/bja/80.2.140

[7] Ayuso MA Sala X Luis M Carbo JM. Predicting difficult orotracheal intubation in pharyngo-laryngeal disease: preliminary results of a composite index. Can J Anaesth. 2003;50:81–85. 12514157 10.1007/BF03020193

[8] Bryan YF Morgan AG Johnson KN. Procedural challenges during intubation in patients with oropharyngeal masses: a prospective observational study. Anesth Analg. 2019;128:1256–1263. 31094797 10.1213/ANE.0000000000004089

[9] Kohse EK Siebert HK Sasu PB. A model to predict difficult airway alerts after videolaryngoscopy in adults with anticipated difficult airways—the VIDIAC score. Anaesthesia. 2022;77:1089–1096. 36006056 10.1111/anae.15841

[10] Law JA Morris IR Brousseau PA Sylvia de la R Milne AD. The incidence, success rate, and complications of awake tracheal intubation in 1,554 patients over 12 years: an historical cohort study. Can J Anaesth. 2015;62:736–744. 25907462 10.1007/s12630-015-0387-y

[11] Rosenblatt W Ianus AI Sukhupragarn W Fickenscher A Sasaki C. Preoperative endoscopic airway examination (PEAE) provides superior airway information and may reduce the use of unnecessary awake intubation. Anesth Analg. 2011;112:602–607. 21081768 10.1213/ANE.0b013e3181fdfc1c

[12] Detsky ME Jivraj N Adhikari NK. Will this patient be difficult to intubate?: the rational clinical examination systematic review. JAMA. 2019;321:493–503. 30721300 10.1001/jama.2018.21413

[13] el-Ganzouri AR McCarthy RJ Tuman KJ Tanck EN Ivankovich AD. Preoperative airway assessment: predictive value of a multivariate risk index. Anesth Analg. 1996;82:1197–1204. 8638791 10.1097/00000539-199606000-00017

[14] Norskov AK. Preoperative airway assessment—experience gained from a multicentre cluster randomised trial and the Danish anaesthesia database. Dan Med J. 2016;63:B5241. 27127020

[15] Roth D Pace NL Lee A. Airway physical examination tests for detection of difficult airway management in apparently normal adult patients. Cochrane Database Syst Rev. 2018;5:CD008874. 29761867 10.1002/14651858.CD008874.pub2 PMC6404686

[16] Wilson ME Spiegelhalter D Robertson JA Lesser P. Predicting difficult intubation. Br J Anaesth. 1988;61:211–216. 3415893 10.1093/bja/61.2.211

[17] Samsoon GL Young JR. Difficult tracheal intubation: a retrospective study. Anaesthesia. 1987;42:487–490. 3592174 10.1111/j.1365-2044.1987.tb04039.x

[18] Collins GS Reitsma JB Altman DG Moons KG. Transparent reporting of a multivariable prediction model for individual prognosis or diagnosis (TRIPOD): the TRIPOD statement. BMJ. 2015;350:g7594. 25569120 10.1136/bmj.g7594

[19] Heus P Reitsma JB Collins GS. Transparent reporting of multivariable prediction models in journal and conference abstracts: TRIPOD for abstracts. Ann Intern Med. 2020;173:42–47. 10.7326/M20-0193 32479165

[20] Kattan MW Vickers AJ. Statistical analysis and reporting guidelines for CHEST. Chest. 2020;158:S3–S11. 32658650 10.1016/j.chest.2019.10.064 PMC8176645

[21] Riley RD Ensor J Snell KIE. Calculating the sample size required for developing a clinical prediction model. BMJ. 2020;368:m441. 32188600 10.1136/bmj.m441

[22] Grensemann J Mohlenkamp E Breitfeld P. Tracheal tube-mounted camera assisted intubation vs. videolaryngoscopy in expected difficult airway: a prospective, randomized trial (VivaOP Trial). Front Med (Lausanne). 2021;8:767182. 34977071 10.3389/fmed.2021.767182 PMC8714897

[23] World Health Organization. Fact sheet: overweight and obesity. World Health Organization 2020.

[24] DeLong ER DeLong DM Clarke-Pearson DL. Comparing the areas under two or more correlated receiver operating characteristic curves: a nonparametric approach. Biometrics. 1988;44:837–845. 3203132

[25] Kattan MW Gerds TA. The index of prediction accuracy: an intuitive measure useful for evaluating risk prediction models. Diagn Progn Res. 2018;2:7. 31093557 10.1186/s41512-018-0029-2 PMC6460739

[26] Landis JR Koch GG. The measurement of observer agreement for categorical data. Biometrics. 1977;33:159–174. 843571

[27] Stachler RJ Francis DO Schwartz SR. Clinical practice guideline: hoarseness (Dysphonia) (update). Otolaryngol Head Neck Surg. 2018;158:S1–S42. 29494321 10.1177/0194599817751030

[28] Baldwa NM. Anticipated difficult airway in ear, nose and throat procedures. Int J Otorhinolaryngol Clin. 2015;7:10–16.

[29] Bergler W Maleck W Baker-Schreyer A. [The mallampati score. Prediction of difficult intubation in otolaryngologic laser surgery by mallampati score]. Anaesthesist. 1997;46:437–440. 9245215 10.1007/s001010050423

[30] Tasli H Karakoc O Birkent H. A grading system for transnasal flexible laryngoscopy. J Voice. 2019;33:712–715. 29730193 10.1016/j.jvoice.2018.02.019

[31] Bastian RW Collins SL Kaniff T Matz GJ. Indirect videolaryngoscopy versus direct endoscopy for larynx and pharynx cancer staging. Toward elimination of preliminary direct laryngoscopy. Ann Otol Rhinol Laryngol. 1989;98:693–698. 2782803 10.1177/000348948909800906

[32] Sartoris A. [Endoscopy in the diagnosis and staging of cancer of the larynx]. Acta Otorhinolaryngol Ital. 1991;11(suppl 33):23–25. 1927503

[33] Yamamoto K Tsubokawa T Shibata K. Predicting difficult intubation with indirect laryngoscopy. Anesthesiology. 1997;86:316–321. 9054250 10.1097/00000542-199702000-00007

[34] Gemma M Buratti L Di Santo D. Pre-operative transnasal endoscopy as a predictor of difficult airway: a prospective cohort study. Eur J Anaesthesiol. 2020;37:98–104. 31789897 10.1097/EJA.0000000000001127

[35] Guo Y Feng Y Liang H. Role of flexible fiberoptic laryngoscopy in predicting difficult intubation. Minerva Anestesiol. 2018;84:337–345. 28984098 10.23736/S0375-9393.17.12144-9

[36] Law JA Duggan LV Asselin M. Canadian airway focus group updated consensus-based recommendations for management of the difficult airway: part 2. planning and implementing safe management of the patient with an anticipated difficult airway. Can J Anaesth. 2021;68:1405–1436. 34105065 10.1007/s12630-021-02008-z PMC8186352

[37] Heidegger T. Management of the difficult airway. N Engl J Med. 2021;384:1836–1847. 33979490 10.1056/NEJMra1916801

[38] Gaszynski T. A comparison of pre-operative transnasal flexible endoscopic laryngoscopy and actual laryngeal view obtained with videolaryngoscopy in predicted difficult intubations. Eur J Anaesthesiol. 2021;38:201–202. 33394801 10.1097/EJA.0000000000001255

[39] Subramanian V Mascha EJ Kattan MW. Developing a clinical prediction score: comparing prediction accuracy of integer scores to statistical regression models. Anesth Analg. 2021;132:1603–1613. 33464759 10.1213/ANE.0000000000005362

[40] Dankert A Dohrmann T Loser B. Pulmonary function tests for the prediction of postoperative pulmonary complications. Dtsch Arztebl Int. 2022;119:99–106. 34939921 10.3238/arztebl.m2022.0074 PMC9131183

