# Supplementary material for: Transnasal Videoendoscopy for Preoperative Airway Risk Stratification: Development and Validation of a Multivariable Risk Prediction Model
Source: Anesth Analg. 2023 Apr 19;136(6):1164–73. doi: 10.1213/ANE.0000000000006418 (PMC10171290; doi:10.1213/ANE.0000000000006418)
Supplement: Supplementary file 1 [file ane-136-1164-s001.docx]

**Supplementary Table S1** Decision thresholds: sensitivity, specificity, positive predictive value, and negative predictive value of the TVE score

| TVE score (points) | Sensitivity  (%) | Specificity  (%) | Positive predictive value (%) | Negative predictive value (%) |
| --- | --- | --- | --- | --- |
| ≥2 | 94.0% | 23.5% | 28.8% | 92.2% |
| ≥3 | 91.1% | 31.9% | 30.6% | 91.6% |
| ≥4 | 85.8% | 41.3% | 32.5% | 89.8% |
| ≥5 | 79.8% | 48.3% | 33.7% | 87.9% |
| ≥6 | 69.5% | 61.1% | 37.1% | 85.9% |
| ≥7 | 62.1% | 67.1% | 38.3% | 84.3% |
| ≥8 | 45.4% | 79.6% | 42.2% | 81.6% |
| ≥9 | 38.3% | 84.8% | 45.4% | 80.7% |
| ≥10 | 31.2% | 88.4% | 47.1% | 79.6% |
| ≥11 | 25.5% | 91.7% | 50.3% | 78.9% |
| ≥12 | 19.9% | 94.5% | 54.4% | 78.2% |
| ≥13 | 13.8% | 96.1% | 54.2% | 77.2% |
| ≥14 | 9.6% | 97.3% | 54.0% | 76.6% |

Values are number (proportion); evaluation of the optimal decision threshold for the TVE score: light grey: ≥7 points was considered a screening cut-off value with a sensitivity >60%; dark grey: a second cut-off value was selected (≥9 points) as a diagnostic cut-off value with a specificity >80%; resulting risk rankings are: 7-8 points (increased risk) and ≥9 points (highest risk)
